# Severe Arterial Thrombosis Revealing Underlying Nephrotic Syndrome

**DOI:** 10.7759/cureus.100741

**Published:** 2026-01-04

**Authors:** Megha Prakash, Arjun Sabharwal

**Affiliations:** 1 Nephrology, Doncaster and Bassetlaw Teaching Hospitals, Doncaster, GBR

**Keywords:** acute arterial thrombosis, adult nephrotic syndrome, focal segmental glomerulosclerosis (fsgs), low albumin, membranous nephropathy, minimal change nephrotic syndrome (mcns), rituximab therapy, thromboembolic events

## Abstract

A man in his 30s with hypertension presented with acute left lower limb pain and numbness for one week. Imaging revealed a thrombus at the left iliac bifurcation, following which he underwent urgent thromboembolectomy. Despite repeated interventions and anticoagulation, re-occlusion occurred the following day, ultimately requiring an above-knee amputation.

Persistent hypoalbuminaemia and proteinuria prompted further evaluation, revealing nephrotic syndrome. Due to heparin resistance from severe hypoalbuminaemia, anticoagulation was switched from unfractionated heparin to argatroban.

Immunosuppression with high-dose steroids and rituximab was initiated, chosen to cover potential causes including minimal change disease (MCD), membranous nephropathy (MN), and focal segmental glomerulosclerosis (FSGS). A renal biopsy could not be performed due to continuous anticoagulation. The second rituximab dose was delayed due to stump infection of the amputated limb. The patient’s albumin and proteinuria improved significantly following the treatment.

This case highlights the need for early urine protein testing in unexplained hypoalbuminaemia or arterial thrombosis, to investigate nephrotic syndrome as a potential cause. Prompt treatment is key to preserve kidney function and reduce the chance of limb loss.

## Introduction

Nephrotic syndrome is defined as having proteinuria (>3.5 g/day), hypoalbuminaemia (<3 g/dL), and clinical signs such as oedema [1]. Thromboembolism is one of the most common complications of nephrotic syndrome in adults. This occurs due to urinary loss of anticoagulant factors such as antithrombin III, platelet hyperactivity, and increased synthesis of procoagulant proteins by the liver [1].

Most of these thrombotic events occur in the venous system, affecting renal veins or pulmonary arteries, whereas arterial thrombosis is much less common. Thrombotic events tend to present after an initial diagnosis of nephrotic syndrome has been made [2], making the presentation outlined in this case unique. Arterial thrombosis as the first presentation of nephrotic syndrome is rare, and when evidenced in existing literature, it is often as a single case [3,4].

It is critical to recognise signs of this complication, as arterial thrombosis can lead to permanent disability, such as limb loss. This report discusses a rare case of nephrotic syndrome presenting initially with acute limb ischaemia and illustrates the diagnostic challenges for nephrologists and vascular surgeons.

## Case presentation

A 39-year-old man with a past medical history significant for only hypertension presented with a one-week history of progressive pain and numbness in his left lower limb. On examination, blood pressure was 146/109, and the limb was cold with absent distal pulses, consistent with Rutherford class I acute limb ischaemia.

**Figure 1:**
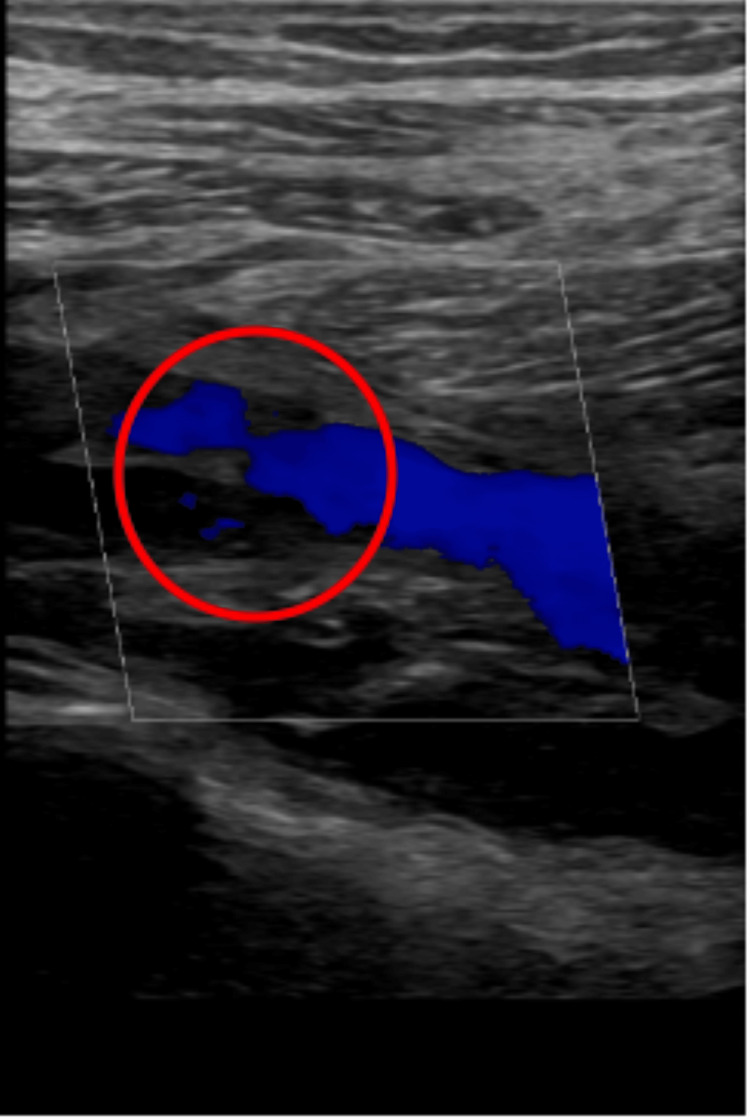
Ultrasound demonstrating occlusion of the left popliteal artery

**Figure 2:**
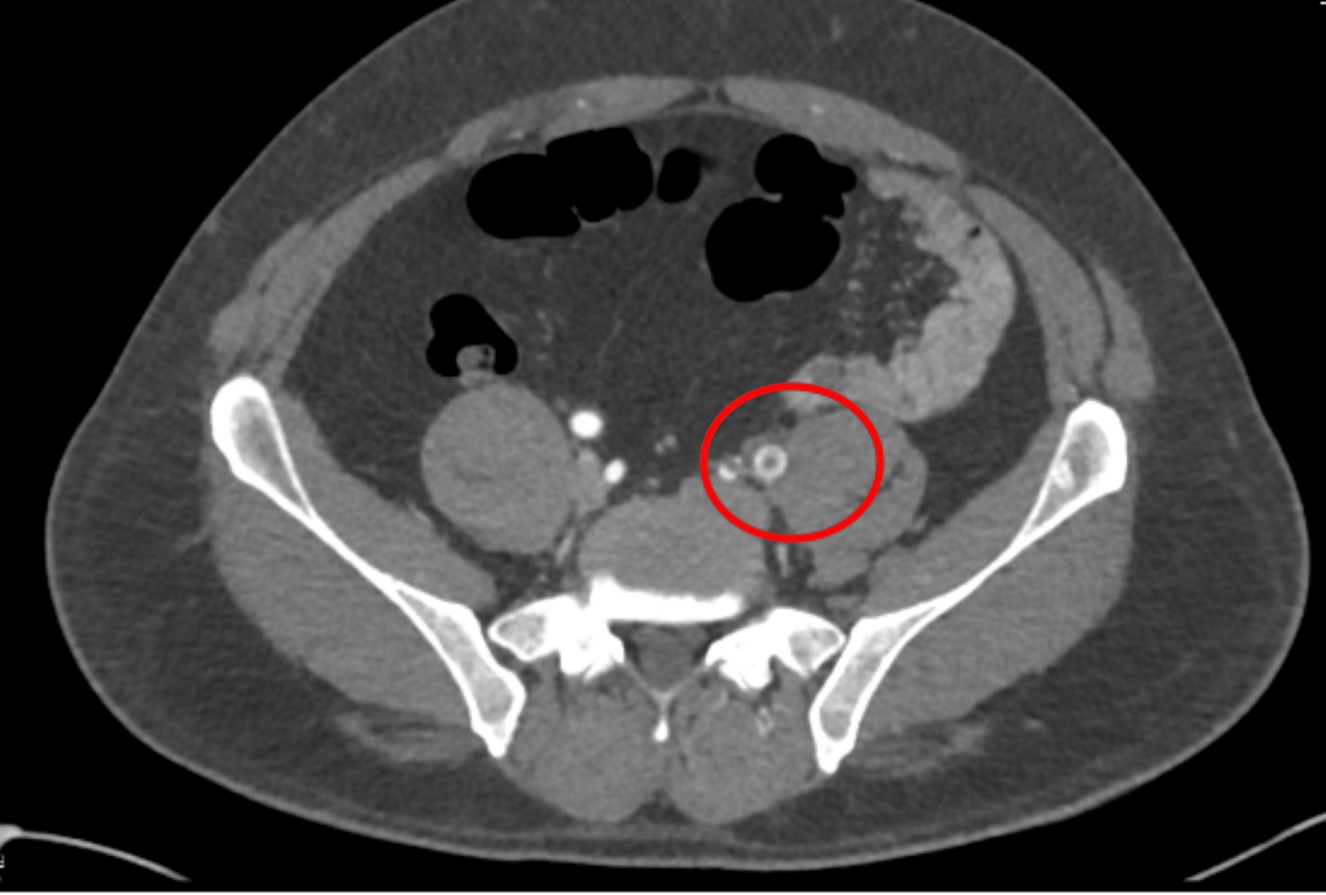
Initial CT angiogram showing a thrombus at the left iliac bifurcation CT: computed tomography

**Figure 3:**
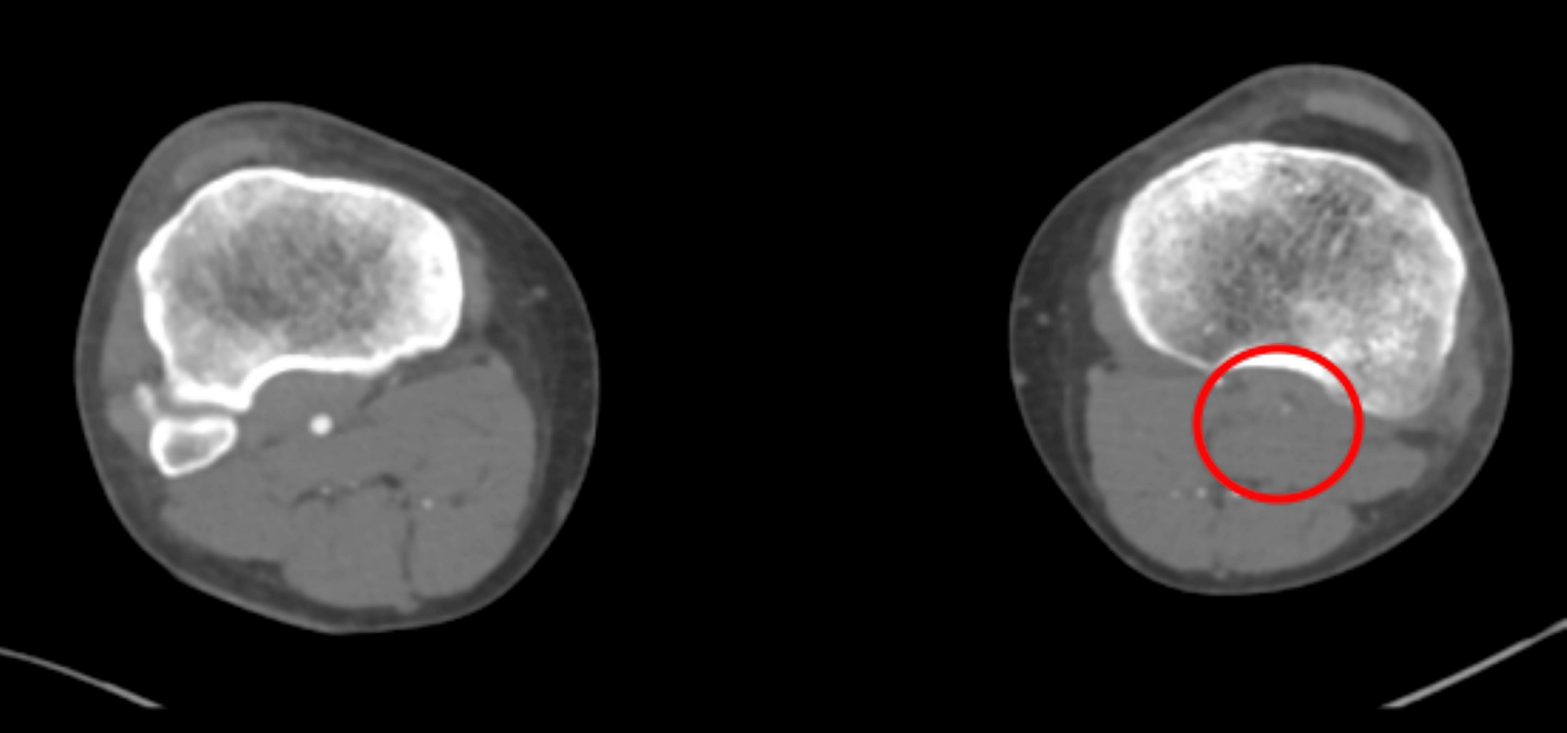
Initial CT angiogram showing left popliteal occlusion CT: computed tomography

An urgent duplex venous ultrasound demonstrated occlusion of the popliteal artery (Figure 1). A computed tomography angiogram revealed a large thrombus at the left iliac bifurcation (Figure 2), with mild popliteal and probable dorsalis pedis occlusion (Figure 3). He was started on an intravenous (IV) unfractionated heparin infusion and admitted under the vascular surgery team, where he underwent an emergency left femoral, popliteal, and selective anterior tibial (AT) and tibioperoneal trunk (TPT) thromboembolectomy on the same day. However, a postoperative Doppler continued to show absent lower limb pulses.

**Figure 4:**
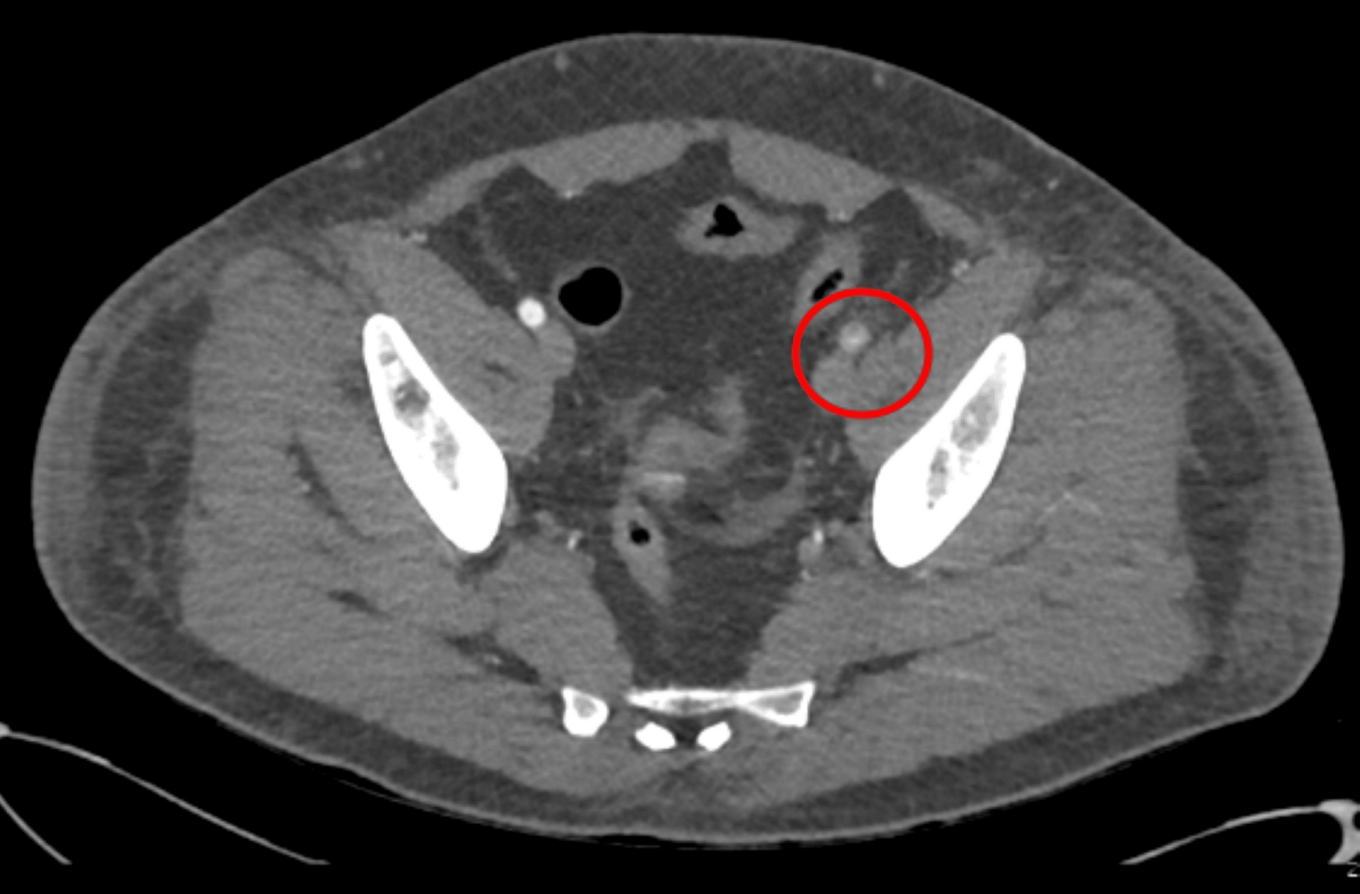
Repeat CT angiogram showing re-occlusion throughout the left limb CT: computed tomography

**Figure 5:**
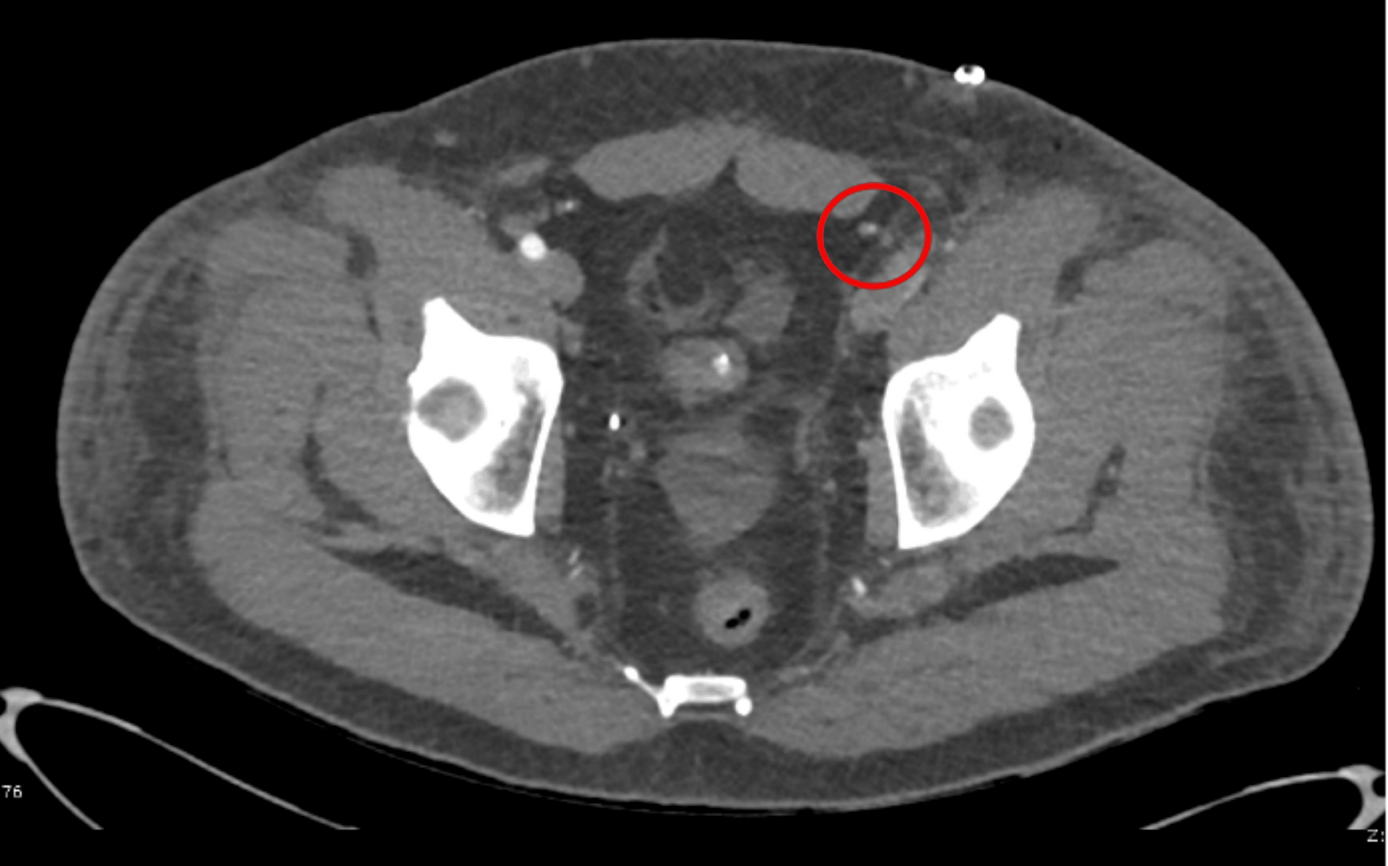
Repeat CT angiogram showing re-occlusion throughout the left limb CT: computed tomography

**Figure 6:**
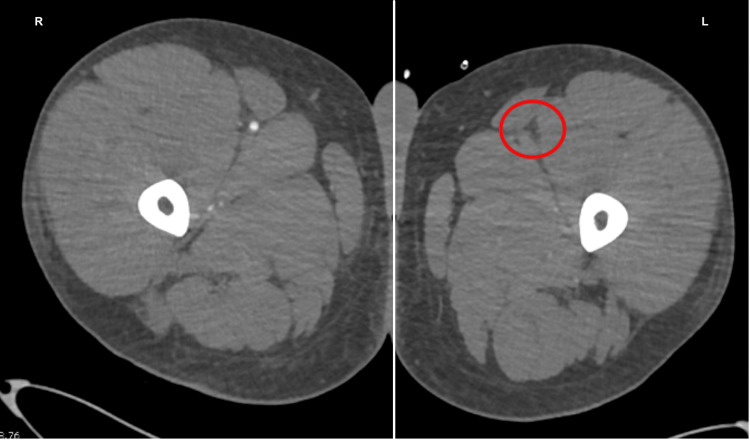
Repeat CT angiogram of both lower limbs showing re-occlusion throughout the left limb CT: computed tomography

**Figure 7:**
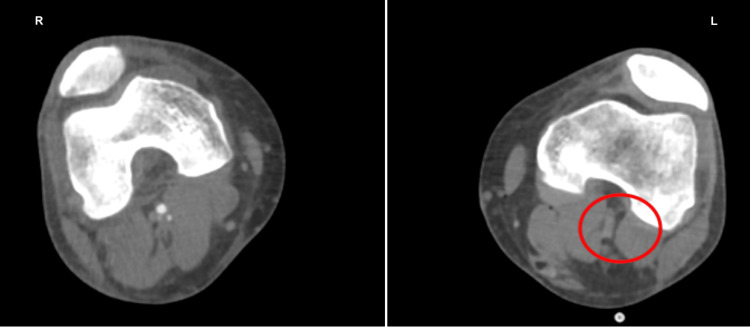
Repeat CT angiogram of both lower limbs showing re-occlusion throughout the left limb CT: computed tomography

A redo emergency thrombectomy was performed by the vascular surgeons the following day, during which a fresh thrombus was noted at the previous site, and an intraoperative Doppler revealed great saphenous vein thrombosis (this was done during the operation; therefore, the image is unavailable). A repeat CT angiogram showed re-occlusion of the left lower limb (Figures 4-7). The sensory and motor deficits remained despite the two attempts to revascularise the limb; therefore, a decision was made the next day to carry out an above-knee amputation.

Initial laboratory investigations showed a low albumin level of 7 g/L and mild acute kidney injury (Table 1), with no other significant findings.

**Table 1:** Albumin and creatinine levels on admission

Parameter	Initial value	Reference range
Albumin (g/L)	7	35-50
Serum creatinine (µmol/L)	120	64-104

During his admission, the patient was reviewed by haematology regarding the repeated thrombotic events despite being on an unfractionated heparin infusion. A marked drop in albumin was noted, which was thought to be impacting the efficacy of heparin and leading to inadequate levels of anticoagulation. The patient still required a continuous infusion of anticoagulation due to the risk of a repeat thrombus; therefore, he was switched to an argatroban infusion and admitted to the intensive care unit.

The patient was then reviewed by the nephrology team regarding the persistent hypoalbuminaemia and found to have proteinuria and a raised urine protein-to-creatinine ratio (Table 2), which led to a diagnosis of nephrotic syndrome. He was discussed in a specialist renal multidisciplinary team (MDT) meeting and started on IV methylprednisolone (500 mg once a day) and given one dose of rituximab.

**Table 2:** Urine investigations The UPCR test measures the amount of protein relative to creatinine in urine, with elevated levels signifying renal damage. UPCR: urine protein-to-creatinine ratio

Parameter	Value	Reference range
Urine protein concentration (g/L)	13.51	0.01-0.14
Urine creatinine concentration (mmol/L)	17.4	5.1-14.2
UPCR (mg/mmol)	776	0-15

Various other laboratory tests (Table 3) were undertaken whilst under the nephrology team to narrow down the differential diagnoses. Although a renal biopsy would have aided in providing a diagnosis, this was not feasible due to the continuous anticoagulation requirements. A genetic screen was also sent; however, the results of this are still awaited.

**Table 3:** Additional immunology screen ANCA: anti-neutrophil cytoplasmic antibody, HIV: human immunodeficiency virus

Parameter	Result	Reference range
Syphilis antibody (treponemal antibody)	Not detected	N/A
HIV 1 + 2 antibody/antigen	Not detected	N/A
Beta-2-glycoprotein IgM and IgG (u/mL)	<1	0-10
Phospholipase A2 receptor antibodies (RU/mL)	<3	0-13
Complement C3 (g/L)	1.58	0.75-1.65
Complement C4 (g/L)	>0.64	0.14-0.54
IgG (g/L)	3	6-16
IgA (g/L)	1.74	0.8-2.8
IgM (g/L)	0.32	0.5-1.9
IgG anti-cardiolipin (GPL-U/mL)	<1	0-40
IgM anti-cardiolipin (GPL-U/mL)	<1	0-40
ANCA	Negative	N/A

Due to a combination of the underlying condition and the immunosuppressant effects of the steroids and rituximab, the patient developed a stump infection of the amputated limb requiring intravenous antibiotics. The second dose of rituximab, which should have been given two weeks following the first, had to be delayed so as not to worsen the infection and was given four weeks after.

Following treatment, the patient’s albumin improved to 37 g/L, and UPCR dropped dramatically from 776 to 37 mg/mmol (Table 4) within one month of admission. Creatinine levels returned to normal, confirming preserved renal function.

**Table 4:** Comparison of albumin, creatinine, and UPCR values on admission to at the time of discharge UPCR: urine protein-to-creatinine ratio

Parameter	Initial value	Peak/lowest value	Value on discharge	Reference range
Albumin (g/L)	7	7	36	35-50
Serum creatinine (µmol/L)	120	159	58	64-104
UPCR (mg/mmol)	776	776	37	0-15

The patient was discharged on a weaning regimen of oral prednisolone and a direct oral anticoagulant (DOAC) for thrombosis prevention. He will require ongoing outpatient follow-up, the first of which was scheduled in 2-4 weeks, and intense rehabilitation due to the loss of the limb.

## Discussion

Thrombosis is a well-documented complication of nephrotic syndrome, with deep vein thrombosis, renal vein thrombosis, and pulmonary embolism being the most common. Not only are arterial thromboses less common, but they also very rarely occur as the initial presentation before a diagnosis of nephrotic syndrome has been made [4].

Arterial thrombosis can lead to very poor outcomes for patients, such as limb loss or stroke. As arterial thrombosis is a rare presentation of nephrotic syndrome, there is limited evidence of similar presentations. A retrospective study from 2023 assessed patients with arterial thrombosis and nephrotic syndrome, discussing nine patients between 2011 and 2023 with both conditions [5]. Of these, three required an above-knee amputation due to lower limb ischaemia despite intervention. This goes to show that although uncommon, when arterial thrombosis does occur, it can lead to very poor outcomes for patients. This is why increasing awareness of the complication is crucial for early detection and improved patient outcomes.

Initially, in our case, the arterial thrombus was thought to be secondary to an atherosclerotic plaque rupture or embolus. However, the combination of severe hypoalbuminaemia, proteinuria, and heparin resistance prompted investigation for nephrotic syndrome as the underlying cause.

The primary diagnostic considerations in this case were as follows: (1) minimal change disease (MCD), possible given preserved renal function, but less likely due to age; (2) membranoproliferative glomerulonephritis (MPGN), a common cause in adults; (3) focal segmental glomerulosclerosis (FSGS), possible, especially given the patient’s age; and (4) membranous nephropathy (MN), a common cause of nephrotic syndrome in adults, may be idiopathic, and is the aetiology most frequently associated with thrombus in nephrotic syndrome [6].

Rituximab was chosen as the treatment as it is effective for all of these differentials, covering all potential causes, and shown to provide greater remission, particularly in younger patients [7,8]. In the circumstance where a non-targeted renal biopsy was not possible due to high thrombotic risk and inability to safely interrupt anticoagulation [9], this remained the best management option in addition to the initial high-dose steroids.

## Conclusions

Nephrotic syndrome can, uncommonly, present initially with arterial thrombosis. As this is a rare and non-specific presentation, it can be challenging to diagnose, and various obstacles arise in its management. However, early recognition leads to improved renal function and patient outcomes, avoiding severe consequences such as limb loss.

Therefore, nephrotic syndrome should always be considered as a differential diagnosis in patients presenting with an arterial thrombosis of unknown cause, and even more so if the patient has risk factors of arterial disease. In such cases, a urine protein-to-creatinine ratio and albumin levels should be checked. In situations where nephrotic syndrome is suspected and multiple pathologies are possible, but it is unsafe to carry out a biopsy, rituximab is a valuable treatment option. This case report increases awareness by describing an uncommon initial presentation of nephrotic syndrome and outlining the impact that delayed recognition can have on the patient, as well as outlining potential challenges involved in treatment.
